# Incidental Finding of Atrial Myxoma in a Patient Presenting With Transient Ischemic Attack (TIA): A Case Report

**DOI:** 10.7759/cureus.61082

**Published:** 2024-05-25

**Authors:** Ujala Akhtar, Hamid Naeem, Sanam Fida, Qazi Muhammad Farooq Wahab

**Affiliations:** 1 Cardiac Surgery, Rehman Medical Institue, Peshawar, PAK; 2 Cardiac Surgery, Rehman Medical Institute, Peshawar, PAK; 3 Internal Medicine, Khyber Teaching Hospital (KTH), Peshawar, PAK

**Keywords:** neurological manifestation, embolic phenomena, cardiac tumour, atrial myxoma, transient ischemic attacks

## Abstract

Atrial myxomas are benign primary cardiac tumors. They can present with nonspecific symptoms, ranging from constitutional symptoms and embolic phenomena such as transient ischemic attacks (TIAs) or strokes to sudden cardiac death. Early diagnosis may be a challenge due to the nonspecific presentation of atrial myxoma. A high degree of suspicion is needed in patients with TIA having no known cardiovascular risk factors. Although benign, if left untreated, it can lead to serious complications ranging from embolic phenomena and obstructive symptoms to sudden cardiac death. An echocardiogram is of fundamental importance in diagnosing atrial myxoma, and surgical resection is the ultimate treatment of choice. Here, we discuss a case of TIA as the initial presentation of atrial myxoma.

## Introduction

Primary cardiac tumors account for 5% of neoplasia, with atrial myxoma being the most common, accounting for 45% to 50% of cases [1]. Approximately 75% of cardiac myxomas occur in the left atrium. They commonly arise from the atrial septum and are gelatinous with a smooth or lobulated surface on gross examination. Clinical manifestations of atrial myxoma vary greatly, and patients usually present a wide range of symptoms, including arrhythmias, embolic phenomena, intracardiac flow obstruction, and constitutional symptoms [2].

Cardiovascular myxoma symptoms are produced by mechanical interference with cardiac function or embolization. These tumors are friable and account for most cases of tumor embolization. Sometimes, small tumor pieces can break off and enter the bloodstream [3]. If this happens they can block an artery in another part of the body such as the brain or the lungs. Embolisms are the most common complication of cardiac myxoma [4], and cerebral embolism may occur before the onset of other symptoms. The diagnosis is not easily established due to the nonspecific nature of symptoms.

Here, we present a case of a 58-year-old man with a transient ischemic attack (TIA) who was found to have an atrial myxoma.

## Case presentation

A 58-year-old man presented with a complaint of an episode of weakness in his upper right arm and dizziness all of a sudden while taking a bath that lasted only for 15 seconds and resolved completely. He also had significant weight loss in the last year. He had no known comorbidities like diabetes or hypertension. He also denied any significant past medical or surgical history. He denied any previous episode of such weakness.

His vital signs were as follows: blood pressure of 120/78 mmHg, heart rate of 98 beats per minute, respiratory rate of 18 breaths per minute, and temperature of 38 degrees Celsius.

**Figure 1:**
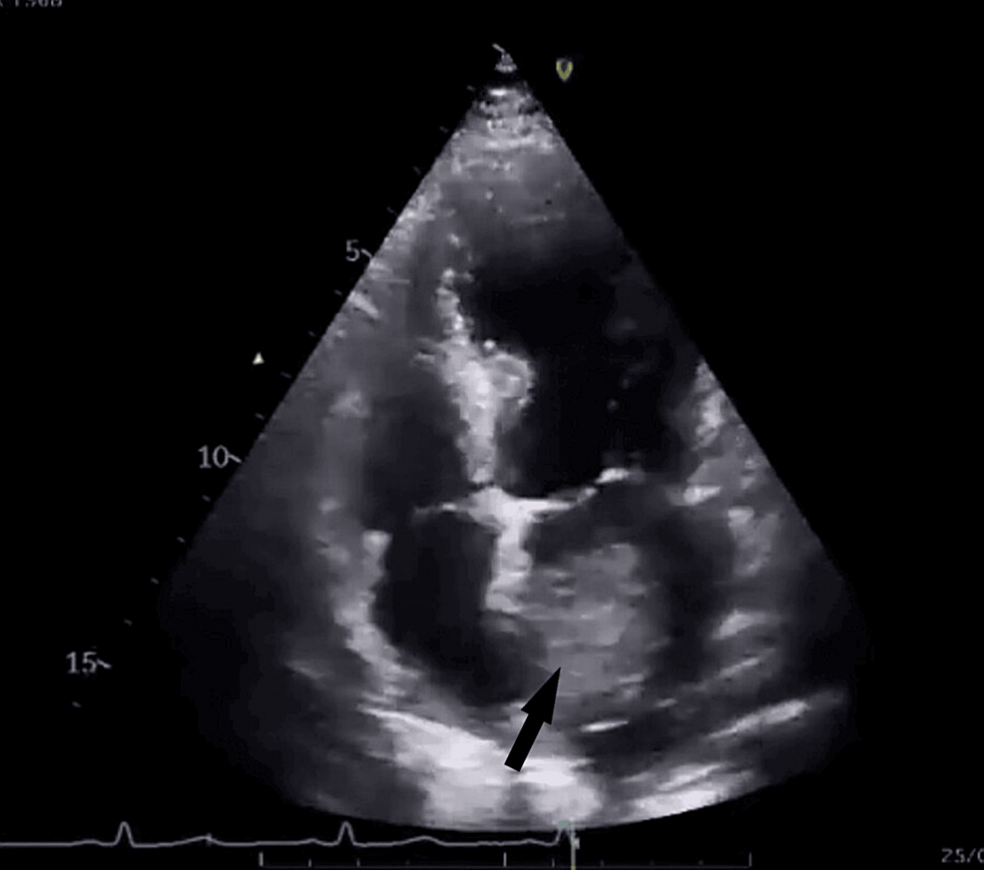
Echocardiogram demonstrates the presence of a left atrial mass, causing left ventricular inflow obstruction.

On initial evaluation, the patient underwent a systemic physical examination, multiple blood tests, and an electrocardiogram (ECG). On cardiovascular examination, there were no murmurs or any significant findings. His ECG revealed normal sinus rhythm. Blood tests revealed normal values on blood count and renal and hepatic functions. To rule out any neurological cause an MRI was performed, which was unremarkable. As part of further evaluation, an echocardiogram was done. The echocardiogram revealed a pedunculated left atrial heterogeneous mass with a lobulated surface attached to the interatrial septum, causing mild to moderate obstruction of the mitral valve during diastole and good biventricular functions (Figure 1).

**Figure 2:**
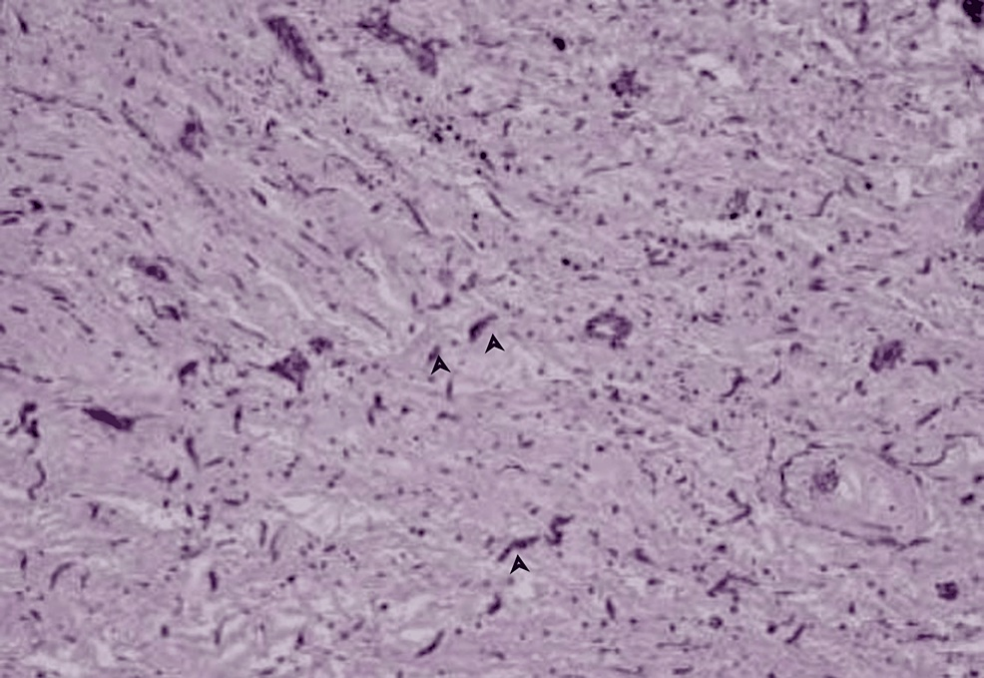
Histopathology showing myxoma cells surrounded by spindles cells, suggesting atrial myxoma.

Before surgical intervention coronary angiogram was done to rule out any neovascularization of the myxoma. He underwent surgery for excision of the left atrial mass and remained symptom-free since then. Specimens were sent for histopathology, which showed myxoma cells surrounded by spindle cells, suggesting atrial myxoma (Figure 2).

## Discussion

Atrial myxoma is frequently present with neurological symptoms in individuals without known cardiovascular risk factors. In the case series by Wen et al., 22 patients were found to have neurological manifestations as initial signs of atrial myxoma [5]. TIA is the most common neurological presentation [6,7].

A case series of 112 cases of left atrial myxoma by Pinede et al. illustrated that this benign tumor may cause a wide range of clinical symptoms not only as cardiac disease but also as neurologic, immunologic, or infective diseases [8]. Early diagnosis may be a challenge due to the nonspecific presentation of atrial myxoma. Prompt diagnosis of the condition can prevent further recurrence and inappropriate anticoagulant therapy. Echocardiography is of fundamental importance in diagnosing atrial myxoma. The transesophageal examination is more accurate than the transthoracic test.

Since myxoma represents an emergency, surgical resection should be performed as soon as possible after the diagnosis is established [9]. Currently, there is no effective medical treatment that arrests the growth of the tumor; thus, early surgical resection of the tumor mass is the best modality of treatment with an excellent prognosis. Resection of tumors not only reduces the recurrence of serious thromboembolic complications but can be curative [3]. As nonspecific symptoms lead to an extensive differential diagnosis, making it difficult to consider atrial myxoma as a cause, it would be prudent to perform echocardiography to rule out cardiac myxoma.

## Conclusions

The presentation of atrial myxoma with nonspecific symptoms delayed its diagnosis, which led to serious consequences. A high degree of suspicion is needed in patients with TIA having no known cardiovascular risk factors. Although benign if left untreated can lead to serious complications ranging from embolic phenomena, obstructive symptoms, to sudden cardiac death. As nonspecific symptoms lead to an extensive differential diagnosis, making it difficult to consider atrial myxoma as a cause, it would be prudent to perform echocardiography to rule out cardiac myxoma.
